# Autosomal recessive woolly hair syndrome: a series of eight patients in an Indian population

**DOI:** 10.1093/skinhd/vzaf082

**Published:** 2025-11-05

**Authors:** Vijay Somani, Anirudh Somani, Ashwini Annabathula

**Affiliations:** Dermatrendz-Dermatology, Hyderabad, Telangana, India; Dermatrendz-Dermatology, Hyderabad, Telangana, India; Dermatrendz-Dermatology, Hyderabad, Telangana, India

## Abstract

Woolly hair is an uncommon disorder of the hair shaft, characterized by tightly coiled scalp hair often accompanied by varying grades of hypotrichosis. Woolly hair involving the entire scalp can be syndromic when associated with various anomalies involving the heart, nervous system, liver, gastrointestinal organs and so on, and is designated as nonsyndromic when it occurs in isolation without any systemic involvement. Nonsyndromic woolly hair can be autosomal dominant or autosomal recessive. We hereby present a series of eight cases of autosomal recessive woolly hair (ARWH) seen in an Indian population in the last 12 years. Their clinical presentation, hair characteristics and response to treatment is described.

What is already known about this topic?

- Woolly hair is an uncommon disorder of the hair shaft, characterized by tightly coiled scalp hair often accompanied by varying grades of hypotrichosis.
- Woolly hair involving the entire scalp can be syndromic or nonsyndromic depending upon systemic involvement.

What does this study add?

- Autosomal woolly hair is an underdiagnosed and under-reported condition.
- This condition appears to be fairly common in India.
- A high index of suspicion, typical clinical characteristics and medical history help with diagnosis.
- Patients typically have healthy-appearing hair at birth, which then fails to grow, usually after a haircut.
- Topical minoxidil 2% twice daily is a safe and effective treatment.

Hair anomalies, often involving the hair shaft, may present with changes in colour, density, length and structure. Autosomal recessive woolly hair (ARWH) is a rare disorder in which patients present with strongly coiled hair on the scalp. The exact prevalence of woolly hair syndrome or its subtypes is not known. The term ‘woolly hair’ was coined by Gossage, when he observed this sign in a European family with an autosomal dominant mode of inheritance.^1^

New research in genomic science has shed more light on the factors that cause woolly hair. Several cellular components, including desmosomal proteins, keratins and signal transduction proteins have been implicated in the formation of woolly hair.^2^ Mutations in various genes are responsible for the phenotypic expression in ARWH. We describe eight patients with nonsyndromic ARWH who presented to our clinic.

The patients had been diagnosed with autosomal recessive woolly hair syndrome in the last 12 years. The age of the patients ranged from 2.5 to 20 years. All were born to nonconsanguineous parents. The pregnancy, perinatal period, growth and development of the patients was unremarkable. The pattern of evolution of the hair disorder was similar in the patients. Normal hair was seen at birth and, after a variable period ranging from 6 months to 1 year, there was an episode of shedding of hair, following which the hair never grew fully. In some cases, hair growth decreased after tonsuring, with increased sparseness and more curling noted after shaving. All patients in the study population had brown-to-black hair except for one who had depigmented hair.

In all the patients, the hair was curled in appearance, thinner, and uniformly short with reduced density and pointed tips. Eyebrows, eyelashes and body hair were normal. A hair-pull test was negative in all except patients 1, 2 and 3. Hair length ranged from 2.5 to 7.5 cm. The patients’ parents were examined for any related anomalies in their skin, hair and teeth. None showed any defect.

Trichograms confirmed a normal anagen-to-telogen ratio in all the patients. Trichoscopy revealed mild variations in the thickness of the hair shafts, and an appearance of crawling snake shafts in some patients. Histopathology was performed in four patients and was essentially unremarkable. None of the patients showed any other abnormality such as palmoplantar keratoderma or keratosis pilaris, or any other hair, teeth or systemic anomaly. Routine investigations, including complete blood count, liver and renal function tests, and chest X-ray, were normal. Normal electrocardiogram and echocardiography results ruled out any cardiac involvement. Genetic testing to corroborate the diagnosis could not be done because of a lack of consent from the patients and financial constraints.

## Discussion

ARWH is an uncommon congenital anomaly, characterized by tightly coiled scalp hairs present at birth or in infancy. The woolly hair phenotype is extremely curly, with an average curl diameter of 0.5 cm.^3^ While the hair growth rate and the anagen-to-telogen ratio were described to be normal, hair often had a smaller diameter and was more fragile than usual.^3^ Patients with woolly hair seem to have a shortened anagen phase despite normal hair growth; therefore, woolly hair does not grow longer than a few centimeters.^4^ Most patients with ARWH suffer from moderate-to-severe hypotrichosis on the scalp. The severity of hypotrichosis varies by patient and family, differs among patients in a given family, or may improve or worsen as patients age. The diagnosis is fairly straightforward and can be made with proper medical history, including family history, clinical examination, a trichogram and a microscopic examination of the hair shafts.

**Figure 1:**
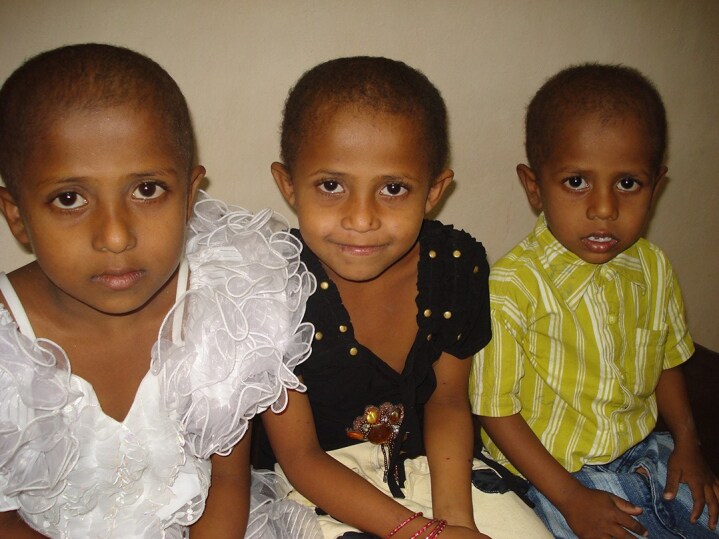
Patients 1, 2 and 3 are siblings who show typical features of autosomal recessive woolly hair syndrome.

**Figure 2:**
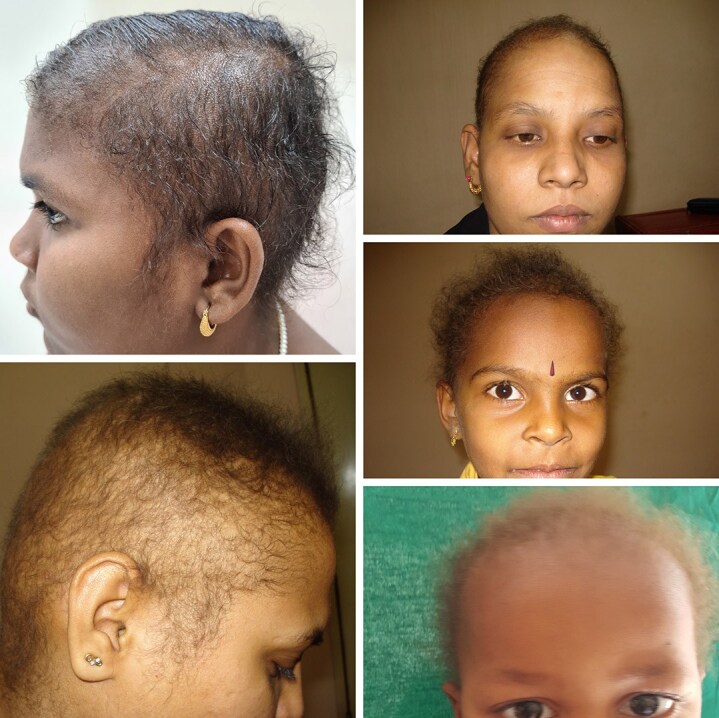
Patients 4, 5, 6, 7 and 8, showing woolly hair.

In this study, we present eight cases of ARWH seen in Indian patients. (Figures 1, 2) All patients in our series had brown-to-black hair, with the lone exception of one patient with blonde hair. All patients in our series presented with generalized woolly scalp hair without any other anomaly. Our patients could be classified as having autosomal recessive woolly hair, based on the lack of woolly hair in other family members, the sporadic occurrence and distinctive clinical features. Of the eight patients, six were girls and two were boys (Table 1).

**Table 1:** Summary of the eight patients with autosomal recessive woolly hair included in this case report

Patient	Age (years)	Sex	Maximum scalp hair length (cm)	Mean growth rate of the hair (mm daily)^a^	Period of normal growth before cessation (months)
1	6	F	2.5	0.3	8
2	4	F	3.0	0.32	6
3	2.5	M	2.5	0.34	12
4	17	F	2.8	0.32	12
5	20	F	3	0.32	Unknown
6	5	F	4.5	0.31	12
7	4	M	4	0.28	Birth
8	12	F	7.5	0.35	Birth

F, female; M, male. ^a^Mean hair growth rate was calculated by shaving an area of approximately 4 cm^2^ at the vertex of the scalp and measuring hair regrowth over the shaved area after 4 weeks.

Several congenital hair shaft disorders that may be considered in the differential diagnosis of ARWH include monilethrix, pili torti, trichorrhexis nodosa, trichorrhexis invaginata and trichothiodystrophy. Hair shafts in monilethrix show a characteristic beaded appearance and often have perifollicular papules and erythema. In pili torti, hair shafts are slightly bent at irregular intervals. Trichorrhesis nodosa and trichorrhexis invaginata show typical hairbrush and bamboo hair deformities. Acquired progressive kinking of the hair typically affects young adults or children and particularly involves the frontotemporal and vertex areas.

ARWH is comprised of several genetically heterogenous groups. Woolly hair autosomal recessive 1 (ARWH1) is caused by mutations in *LPAR6*.^5^ Woolly hair autosomal recessive 2 (ARWH2) is known to be caused by mutations in *LIPH*.^6^ In addition, woolly hair autosomal recessive 3 (ARWH3) is due to mutations in *KRT25*. There is no documented effective treatment for woolly hair and various treatment regimens have been tried. Minoxidil is believed to improve the blood supply to the hair follicles and stimulate hair growth. Topical minoxidil shortens telogen, causing premature entry of resting hair follicles into anagen and also causes prolongation of anagen and increases hair follicle size.

**Figure 3:**
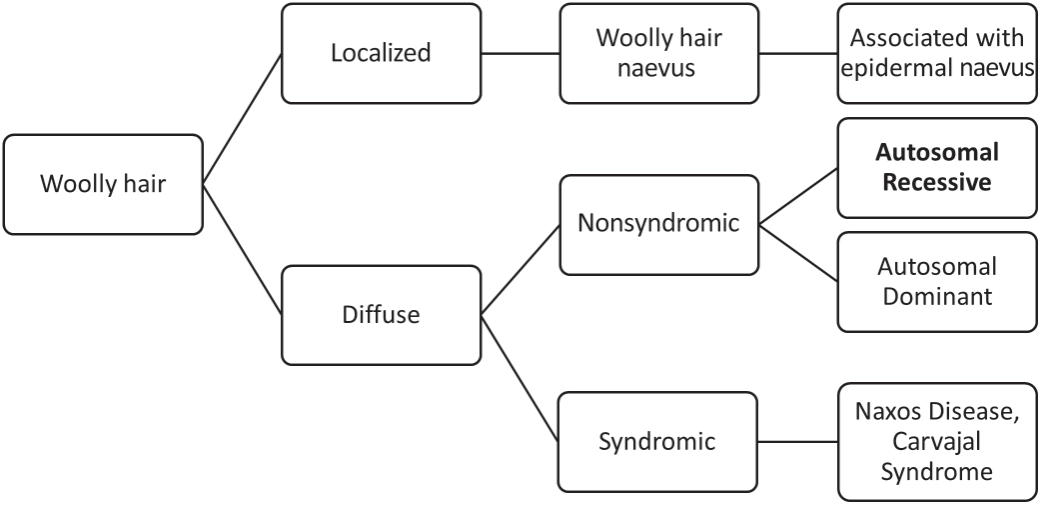
Woolly hair, along with different clinical presentations, can be a presenting sign in several well-defined syndromes.

Localized woolly hair or woolly hair naevus is a benign condition without any associated disorder. Woolly hair, along with different clinical presentations, can be a presenting sign in several well-defined syndromes. These include Naxos syndrome, which is caused by heterozygous mutation in desmoplakin gene; Carvajal-Huerta syndrome, which is due to homozygous mutation in desmoplakin protein; the woolly hair/hypotrichosis; ectodermal dysplasia–skin fragility, which is caused by mutation in *PKP1*; and tricho-hepato-enteric syndrome, which is caused by a mutation in *SKIC2*. The triad of woolly hair, arrhythmogenic right ventricular cardiomyopathy and palmoplantar keratoderma (PPK) constitute the autosomal recessive Naxos syndrome. In Carvaja–Huerta syndrome, woolly hair and PPK are associated with left ventricular dilated cardiomyopathy. Therefore, cardiac assessments are crucial in managing these two syndromes. The presence of PPK in a patient with woolly hair should lead the treating physician to rule out any cardiac involvement so that serious consequences are avoided. Woolly hair may present with various features, such as skin fragility, PPK, hypohidrosis, nail dystrophy, cheilitis, abnormal dental development and a desquamating erythematous rash, in patients with ectodermal dysplasia–skin fragility syndrome. Tricho-hepato-enteric syndrome manifests with severe infantile diarrhoea, failure to thrive, dysmorphism, immune or hepatic dysfunction, and trichorrhexis nodosa, in addition to woolly hair. Nonsyndromic woolly hair syndrome is generally associated with hypotrichosis and lighter-coloured, short hair (Figure 3).

Considering that small hair shaft diameters and truncated anagen phase are the main factors responsible for hypotrichosis in ARWH, it is reasonable to assume that minoxidil will be of benefit in patients with ARWH.

Several reports have shown the efficacy of topical minoxidil 1−5% in patients with woolly hair.^7^ Choi *et al*.^8^ reported an increase in hair thickness and density with topical minoxidil 3% and tretinoin 0.025% with oral vitamin D analogue after 5 months of treatment.

**Figure 4:**
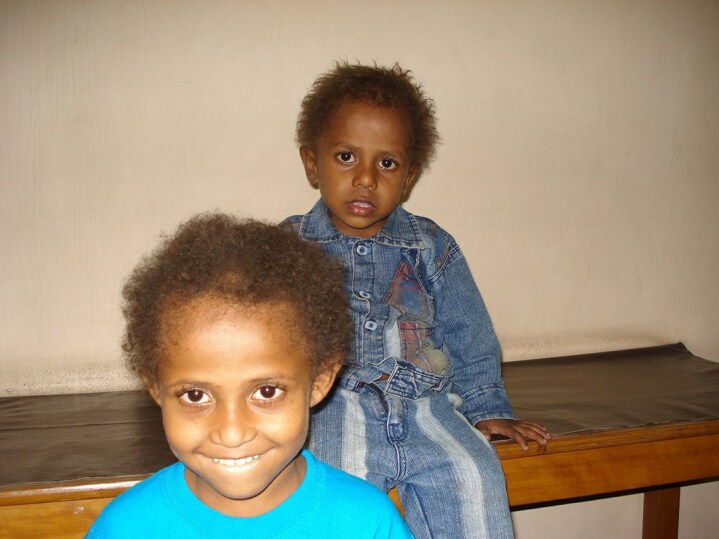
Response to topical minoxidil therapy.

In this series we used minoxidil 2% twice daily in all eight patients, for 6 months, with n increase in hair density, thickness and length noted in six patients (Figure 4). Two patients were lost to follow-up after 2 months.

Another study reported that nonablative fractional lasers induced the growth of intact hair in three adult patients with ARWH.^9^ Recently, four patients with hypotrichosis simplex of the scalp were treated successfully with topical gentamycin.^10^ They showed that gentamicin induced *in vitro* read-through activity across a *CDSN* mutation that causes hypotrichosis simplex. Similarly, gentamycin may be a candidate in the treatment of ARWH via its possible read-through activity in nonsense mutations in *LIPH*, *LPAR6* or *C3orf52*.

## Data Availability

The data underlying this article will be shared on reasonable request to the corresponding author.
